# Diversity and geochemical community assembly processes of the living rare biosphere in a sand-and-gravel aquifer ecosystem in the Midwestern United States

**DOI:** 10.1038/s41598-019-49996-z

**Published:** 2019-09-17

**Authors:** Kyosuke Yamamoto, Keith C. Hackley, Walton R. Kelly, Samuel V. Panno, Yuji Sekiguchi, Robert A. Sanford, Wen-Tso Liu, Yoichi Kamagata, Hideyuki Tamaki

**Affiliations:** 1 Bioproduction Research Institute, National Institute of Advanced Industrial Science and Technology (AIST), Tsukuba, Ibaraki, Japan; 2 Faculty of Life and Environmental Sciences, University of Tsukuba, Tsukuba, Ibaraki, Japan; 3 Isotech (a Stratum Reservoir Brand), Champaign, IL USA; 4 Groundwater Science Section, Illinois State Water Survey, Prairie Research Institute, University of Illinois at Urbana-Champaign (UIUC), Champaign, IL USA; 5 Illinois State Geological Survey, Prairie Research Institute, UIUC, Champaign, IL USA; 6 Biomedical Research Institute, AIST, Tsukuba, Ibaraki, Japan; 7 Department of Geology, UIUC, Urbana, IL USA; 8 Department of Civil and Environmental Engineering, UIUC, Urbana, IL USA; 9 Biotechnology Research Center, The University of Tokyo, Tokyo, Japan

**Keywords:** Microbial ecology, Water microbiology

## Abstract

Natural microbial communities consist of a limited number of abundant species and an extraordinarily diverse population of rare species referred to as the rare biosphere. Recent studies have revealed that the rare biosphere is not merely an inactive dormant population but may play substantial functional roles in the ecosystem. However, structure, activity and community assembly processes of the rare biosphere are poorly understood. In this study, we evaluated the present and living microbial community structures including rare populations in an aquifer ecosystem, the Mahomet Aquifer, USA, by both 16S rDNA and rRNA amplicon deep sequencing. The 13 groundwater samples formed three distinct groups based on the “entire” community structure, and the same grouping was obtained when focusing on the “rare” subcommunities (<0.1% of total abundance), while the “abundant” subcommunities (>1.0%) gave a different grouping. In the correlation analyses, the observed grouping pattern is associated with several geochemical factors, and structures of not only the entire community but also the rare subcommunity are correlated with geochemical profiles in the aquifer ecosystem. Our findings first indicate that the living rare biosphere in the aquifer system has the metabolic potential to adapt to local geochemical factors which dictate the community assembly processes.

## Introduction

Enormous species diversity is a key feature of natural microbial communities and the origin of diversity and its contribution to community function have been central issues of microbial ecology. Microbial communities generally consist of a limited number of abundant species and a vast number of rare species^1^, which generates a “long-tailed” rank-abundance curve. Populations of rare species (e.g. <0.1% of total abundance) in the community, termed as the rare biosphere^2^, are known to be phylogenetically and functionally diverse and redundant, whereas their biological activities have previously been assumed to be lower than those of abundant and proliferated populations^3^. For this reason, it has been considered that most of the rare biosphere remain in a dormant or metabolically inactive state and has a role as a “seed bank”, conferring functional plasticity, robustness, and resilience to the community when subjected to changes in environmental conditions^3,4^. Conversely, an increasing number of studies suggest that the rare biosphere harbors active populations which play substantial roles in community functions despite their low relative abundances^5–8^. Thus, the activity and function of the rare biosphere vary by environments, and its contribution to the entire community structure and function is still controversial^9^.

The microbial community assembly process is highly governed by the chemical profile of habitat which dictates growth conditions for each species and thus deterministically selects community members^10^. Indeed, the relationship between community structure and geochemistry has been investigated in various natural environments, and the geochemical profile has been described as an important driving force for community assembly processes^11,12^. However, most previous studies have focused on overall community or abundant populations, and very little is known whether such a geochemistry-community structure relationship can be seen even in the rare biosphere^13^. In particular, although some recent works highlighted the contribution of deterministic factors to community assembly process of the rare biosphere^14–16^, no reports have investigated and identified key geochemical profiles involving it.

The objective of this study was to gain insights into the community assembly processes of the rare biosphere as well as the abundant biosphere by (1) clarifying the living microbial community structure (rRNA-based amplicon sequencing) including taxa having less than 0.1% of total abundance, (2) obtaining the habitat geochemical information, and (3) evaluating the contribution of various geochemical variables to the community differentiation by correlation analyses. To this end we investigated the bacterial and archaeal community diversity and geochemistry of a subsurface groundwater ecosystem. In subsurface groundwater systems, the relevant microbial communities are highly involved in not only local geochemical cycling but also ecosystem functioning and service such as freshwater supply by removing contaminants which are undesirable for human usage^17^. However, the living rare biosphere in aquifer ecosystems has not been characterized yet, and the activity, function, and roles of them remain largely unknown. The study site was the Mahomet Aquifer, Illinois, USA, a large sand-and-gravel aquifer in east-central Illinois providing drinking water to about 800,000 people. Although there are areas of the aquifer with high levels of arsenic, for the most part the water quality is very good^18^. A unique feature of this site is that the aquifer has a range of environments with hydraulic and geochemical gradients and a variation of geochemical properties due to changes in groundwater chemical variables (e.g., redox conditions, salinity, sulfate, and organic substances) during slow but constant flow within the aquifer^18,19^, making this site suitable for evaluating the relationship between varying geochemical conditions and reacting microbial community structure. These variations in the geochemical environment of the aquifer are due to differences in the structural, lithologic and associated hydrogeology of the Pennsylvanian bedrock. Specifically, an upwelling of fresh groundwater passing from deeper carbonate bedrock and into and through shale bedrock located in the northeastern part of the aquifer has resulted in elevated SO_4_ concentrations (up to 900 mg/L). Similarly, an upwelling of saline groundwater along a geologic structure in shale bedrock at the central-western boundary of the aquifer has resulted in elevated Cl^−^ concentrations (up to 500 mg/L), stronger reducing conditions and associated methane^18,19^.

On the basis of the hypothesis that the local geochemistry highly involves community assembly processes of the rare biosphere, we conducted a deep sequencing analysis of both 16S rRNA and rRNA gene by Illumina MiSeq system for 13 groundwater samples to capture the overall community structure of living microbial populations including the rare biosphere (the rare subcommunity). At the same time, the relationship between groundwater geochemical profiles and microbial community compositions was also evaluated to identify key parameters involving the community assembly process for both the entire community and the rare subcommunity.

## Results

### Population size of subsurface groundwater microbial community

**Figure 1:**
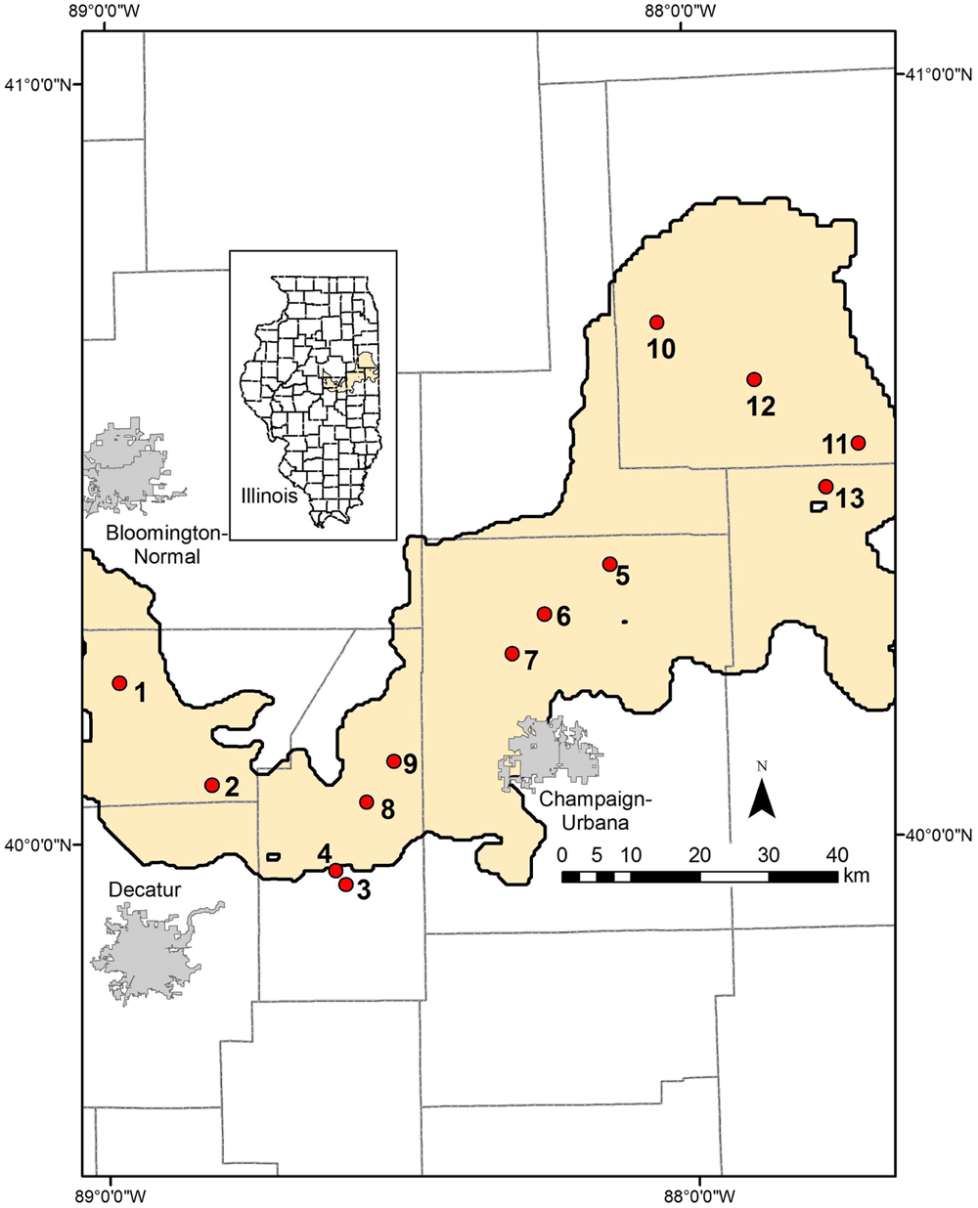
Location of wells sampled in this study. Yellow shading shows extent of the Mahomet Aquifer. Gray lines are country boundaries.

A 16S rDNA and rRNA amplicon sequencing analysis gained 1,907,675 reads from 26 samples (DNA and RNA samples from 13 sampling points; Fig. 1) after quality filtering, and the read number for each sample ranged from 36,322 to 133,990 (Supplementary Table S1). Total cell numbers in each groundwater sample ranged from 3.4 ± 0.9 × 10^5^ cells/mL to 1.2 ± 0.2 × 10^7^ cells/mL (Supplementary Table S1), exhibiting cell densities typical or slightly higher than those in groundwater from uncontaminated aquifers (10^4^–10^6^ cells/mL^20^).

### Community composition in rare subcommunity and entire community

**Figure 2:**
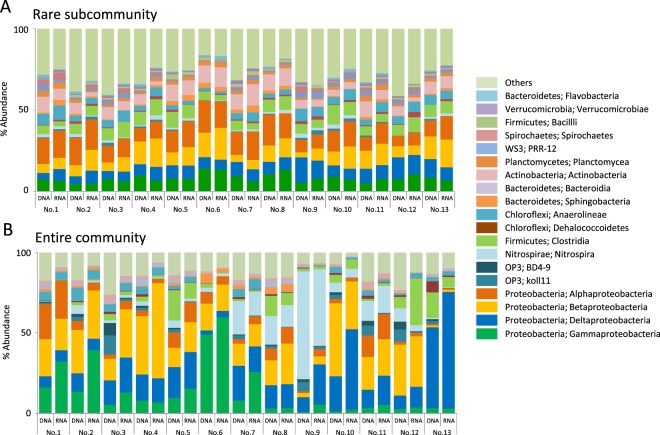
Bacterial community structure of (**A**) the rare subcommunity and (**B**) the entire community of groundwater samples. The OTUs were classified at the species level (97% sequence similarity). The data shown were binned at the class level.

A diverse set of bacterial and archaeal clades were detected in all samples. The structure of rare subcommunity was evaluated and compared with that of entire community. The datasets used for rare subcommunity included clades whose abundance was less than 0.1% in each sample. In the DNA-based rare subcommunity, *Alphaproteobacteria* (12%; average of abundance in each sample), *Betaproteobacteria* (10%), *Deltaproteobacteria* (8%), *Gammaproteobacteria* (8%), *Clostridia* (7%), and *Actinobacteria* (7%) were widespread bacterial taxa (Fig. 2A). These dominant bacterial taxa were likely to evenly distribute among samples. The same trend was also observed in the RNA-based community structure, and the RNA-based proportion of each taxa within a sample did not drastically differ from the DNA-based proportion. In the entire community, proteobacterial classes, *Nitrospira* and *Clostridia* were observed to dominate as was seen for the rare subcommunity (Fig. 2B; *Betaproteobacteria* [20%; abundance in total bacterial population], *Deltaproteobacteria* [16%], *Nitrospira* [11%], *Gammaproteobacteria* [10%], *Alphaproteobacteria* [6%], and *Clostridia* [6%]). In contrast to the rare subcommunity, distribution of these abundant taxa among samples varied by taxon (e.g. *Gammaproteobacteria* tended to be dominant in central and western samples, and *Deltaproteobacteria* were dominant in northeastern samples) (Fig. 2B). The difference between the DNA-based and the RNA-based bacterial community structures was, though it was not drastic, clearer in the entire community compared to that in the rare subcommunity.

**Figure 3:**
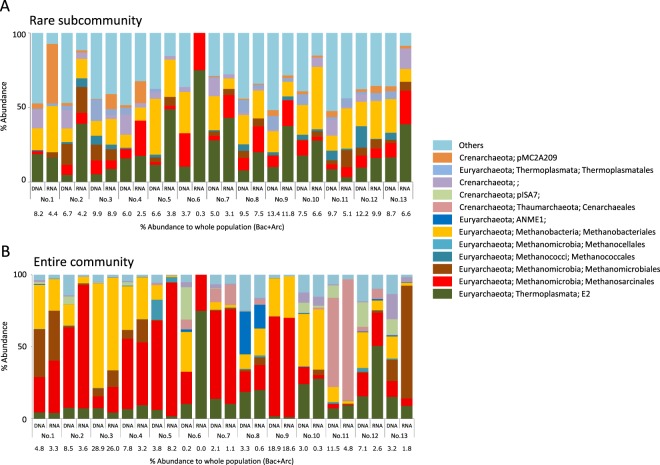
Archaeal community structure of (**A**) the rare subcommunity and (**B**) the entire community of groundwater samples. The OTUs were classified at the species level (97% sequence similarity). The data shown were binned at the order level.

The rare archaeal populations were dominated by E2 (*Thermoplasmata*; 22% average of abundance in each sample) and methanogenic clades e.g. *Methanobacteriales* (18%), *Methanosarcinales* (10%), and *Methanomicrobiales* (5%) (Fig. 3A). In the entire community, methanogenic clades such as orders *Methanobacteriales* (35%; abundance in total archaeal population), *Methanosarcinales* (30%) and *Methanomicrobiales* (4%) predominated in most samples, and a sum of these methanogenic populations comprised up to 70% of total archaeal population of all samples (Fig. 3B). The proportion of archaeal population in each sample ranged from 0.3 to 13.4% (rare subcommunity) and 0 (0.01%) to 29% (entire community) (Fig. 3). In contrast to the bacterial community, the difference between the DNA-based and the RNA-based archaeal community structures was clearer in the rare subcommunity.

Notably, both the rare bacterial and archaeal populations harbored much diverse lineages, which were represented by high abundance of “Others” including a diverse set of functionally-unknown clades (27% and 33%, respectively; Fig. 2A and Fig. 3A), and the proportion of “Others” was greater than those observed in bacterial and archaeal communities in the entire community (13% and 7%, respectively; Fig. 2B and Fig. 3B).

The ratio of RNA- and DNA-based relative abundances is frequently used as an index of living populations (e.g.^21^). Slopes of linear regression of RNA/DNA plots were almost one, and plots of major clades were near the regression lines for both the rare subcommunity and the entire community (Supplementary Fig. S1), indicating that the DNA-based community profile captured living populations in both the rare subcommunity and the entire community.

### Comparison of subsurface groundwater community structure

**Figure 4:**
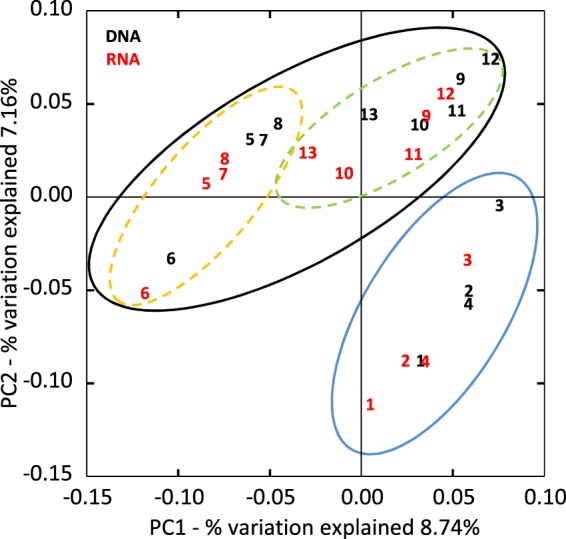
Beta diversity of groundwater communities. Community resemblance was analyzed and visualized by PCoA plot based on unweighted UniFrac distance matrix. Sample number in black indicates DNA-based communities. Sample number in red indicates RNA-based communities. Group I samples are delineated by blue circle. Group II (dashed orange circle) and Group III (dashed green circle) samples are delineated by black circle.

Resemblance of the community structure (beta diversity) among all DNA and RNA samples of groundwater was evaluated by principal coordinate analysis (PCoA) based on unweighted UniFrac distance matrix^22^. UniFrac distance clearly showed a resemblance between DNA- and RNA-based community structures in each sample, indicating that the DNA-based community profile did not capture dead populations but living populations (Supplementary Fig. S2), which is consistent with the pattern observed in the RNA/DNA plots (Supplementary Fig. S1). The 2D-plot of PCoA showed two clusters of the samples (Fig. 4); one was comprised of samples 1 to 4 (Group I: samples from the western part of the aquifer) and the other included the remaining samples. The latter cluster was divided into two subclusters corresponding to geographic region with one exception (Sample 9); one subcluster was comprised of samples 5 to 8 (Group II: samples from the central part) and the other included samples 9 to 13 (Group III: sample 9 plus samples from the northeastern part). The significance of community difference among these three groups was evaluated by the analysis of similarity (ANOSIM) test based on unweighted UniFrac distance matrix. The results supported that the differences between Group I, II and III were significant (Group I vs Group II, R = 0.876, P = 0.001; Group I vs Group III, R = 0.449, P = 0.001; Group II vs Group III, R = 0.561, P = 0.001).

### Subsurface groundwater type defined by geochemical and isotopic parameters

**Figure 5:**
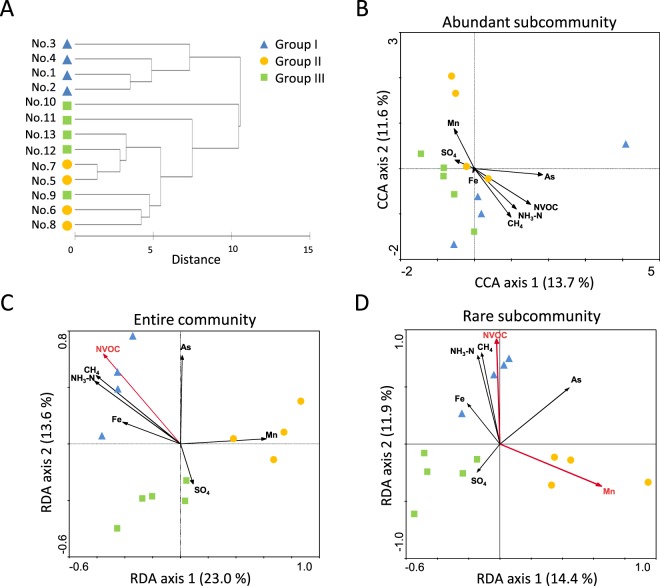
(**A**) Cluster analysis of geochemical and isotopic profiles of groundwater samples. Variables used for the analysis are listed in Table S2. Cluster analysis was applied to matrix of pairwise comparison between samples based on Euclidean distance after data normalization and performed by complete linkage method. (**B**–**D**) CCA and RDA ordination relating community composition with environmental variables. Detrended correspondence analysis was performed with species data prior to CCA and RDA in order to select appropriate analysis. Species and environmental data were analyzed by RDA for the abundant subcommunity and by CCA for the entire community and the rare subcommunity. Red arrows indicate statistically significant variables (P < 0.05).

The geochemical profile of all groundwater samples was analyzed (Supplementary Table S2), and similarity in geochemical profiles between groundwater samples was evaluated by cluster analysis. A strong correlation between geographical location and groundwater type based on geochemical and isotopic profiles was found in our samples. Samples 1 to 4 exhibited similar chemical profiles and formed a cluster distinct from the other samples (Fig. 5A). This groundwater type was characterized by relatively high concentrations of chloride, methane, and non-volatile organic carbon (NVOC), negligible concentration of sulfate, and relatively heavy δ^13^C values of dissolved inorganic carbon (DIC) (Supplementary Table S2). The remaining samples (Samples 5 to 13) exhibited variable chemical profiles but were characterized by relatively high sulfate concentrations and light δ^13^C values of DIC. This geographic distribution of methane-rich western samples (No. 1 to No. 4) and sulfate-rich central and northeastern samples (No. 5 to No. 13) was consistent with previous sampling in the Mahomet Aquifer system^18,19^. This result matched the wide distribution of methanogenic archaea in western region and *Deltaproteobacteria* including sulfate-reducing clades in central and northeastern regions, respectively, based on not only DNA but also RNA datasets (see Discussion and Supplementary Fig. S3). The observed sample grouping pattern here well corresponded to those based on community similarity (Fig. 4), indicating a strong link between geochemical profiles and community structures.

### Comparison of community structure in the abundant and the rare subcommunities

As shown above, the sample clustering based on the geochemical profile was partly correlated with the clustering based on the community structure (Groups I, II and III), implying that geochemical factors affect the community assembly process. To further evaluate beta diversity of the subsurface groundwater community at subcommunity-level and identify geochemical parameters contributing to the microbial community assembly, a correlation between various geochemical variables and the community composition was assessed by redundancy analysis (RDA) and canonical correlation analysis (CCA) (Fig. 5B–D). The datasets used for abundant and rare subcommunities included taxa whose abundance in the community was greater than 1% and less than 0.1% in each sample, respectively. Proportions of abundant and rare populations to entire number of sequence reads in each sample ranged from 58 to 83% (mean value, 68%; SD = 7%), and from 6 to 11% (mean value, 8%; SD = 2%), respectively, and the numbers of taxa in abundant and rare populations ranged from 10 to 27 (median value = 17) and from 305 to 516 (median value = 409), respectively. 2D-plots showed that the samples were divided into three clusters in the entire community and the rare subcommunity, although this trend of clustering was less clear in the abundant subcommunity (Fig. 5B–D). The significance of sample clustering was also supported by ANOSIM testing and Pearson correlation-based cluster analysis (Supplementary Fig. S4). Thus, the subsurface groundwater communities were clearly structured and clustered into groups even at the level of the rare subcommunity.

### Correlation between community structure and geochemical parameters

On a 2D-plot of the entire community (Fig. 5C), a cluster of the Group I samples (western samples) was highly correlated with NVOC, methane, ammonium, and iron. The Group II samples (central samples) were correlated with manganese, and the Group III samples (northeastern samples plus No. 9) were mainly associated with sulfate. Overall trends of correspondence between geochemistry and community structure were observed; direction of most arrows tended to separate Group I from Groups II and III, but the arrows directed for separating Group II from Group III were less clear. Notably, a 2D-plot of the rare subcommunity (Fig. 5D) exhibited a similar trend to that of the entire community, although the abundant subcommunity (Fig. 5B) exhibited less clear trends in both sample ordination and its correspondence with geochemical parameters than those of the rare subcommunity and the entire community. These results indicate that the community assembly of not only the entire community but also the rare biosphere reflects the environmental geochemical factors.

### Taxa contributing to community differentiation

The contribution of each bacterial and archaeal taxon to the differentiation of the community groups (Groups I, II, and III) was evaluated by similarity percentages (SIMPER) analysis. High-contributing taxa, which ranked in the top 30% of the cumulated contribution to the similarity among the groups were picked up from the entire community, the abundant subcommunity and the rare subcommunity, and the abundance of these taxa in each sample was visualized in heat maps associated with results of the cluster analysis (Supplementary Fig. S5). As for the entire community and the rare subcommunity, the samples were clearly clustered into three groups, and the high-contributing taxa were also clustered into several groups having similar distribution patterns. On the other hand, the samples were not clearly clustered into the groups in the abundant subcommunity. The high-contributing taxa detected in SIMPER analysis in each community were binned at class and order level, and their cumulative contribution to group differentiation are shown in bubble charts in Supplementary Figs S6 and S7.

As for the rare subcommunity, *Alphaproteobacteria* and *Gammaproteobacteria* were characteristic to Group I and II compared to Group III, which can comprise main heterotrophic clades in Group I and II with others (e.g. *Actinomycetales* and *Burkholderiales*). On the other hand, heterotrophic clades characteristic to Group III were *Verrucomicrobia*, *Spirochaetales*, and *Burkholderia*. Group III was also characterized by a dominance of clades harboring diverse obligate anaerobic species such as fermentative bacteria and sulfate-reducing bacteria (e.g. *Syntrophobacterales*, *Clostridiales*, and *Desulfovibrionales*) (Supplementary Figs S6 and S7).

As for the entire community, characteristic taxa of Group I were *Alphaproteobacteria*, *Anaerolineae*, and methanogenic archaea. Characteristic taxa of Group II were *Alphaproteobacteria*, *Betaproteobacteria*, *Gammaproteobacteria*, *Clostridia*, and *Sphingobacteria*. For Group II, features distinguishing this group from the other two groups were a relatively large proportion of *Sphingobacteria* and an absence of *Methanobacteria* and *Anaerolineae*. Characteristic taxa of Group III were *Deltaproteobacteria* and *Nitrospira*. A large proportion of *Deltaproteobacteria*, a small number of *Alphaproteobacteria* and *Gammaproteobacteria*, and an absence of *Verrucomicrobiae* and *Sphingobacteria* were features distinguishing Group III from the other two groups (Supplementary Figs S6 and S7). Thus, the clades characteristic to each group were partly matched between the entire community and the rare subcommunity: less contribution of *Alphaproteobacteria* and *Gammaproteobacteria* and greater contribution of *Deltaproteobacteria* in Group III than in other two groups. In accordance with the community structure, more diverse taxa were identified as contributing taxa in the rare community (increase of “Others”).

## Discussion

The diverse rare biosphere has previously been considered an inactive “seed bank” population, whereas recent works have revealed the existence of a rare-but-active population by focusing on specific functional guilds or metabolic processes (reviewed in 1, 9). Hence, to evaluate the activity of a rare population it is crucial to expand our knowledge of diversity and function of rare biosphere in various natural environments. The 16S rRNA and rDNA amplicon deep sequencing results obtained in the present study show that rare populations in the groundwater community are not just dead cells but rather alive. Although limitations about using RNA/DNA ratio as an index of cellular metabolic activities has been pointed out^23^, recent studies that evaluated methods to detect living population^24,25^ have suggested that it can still be useful with care to estimate living members, not dead cells or extracellular DNA within a community harboring diverse phylogenetic and functional groups. We compared the abundance of each clade between the DNA- and the RNA-based community and found that most clades in the rare subcommunity did not show a highly differential abundance (Figs 2 and 3), indicating that the activity of each rare subcommunity member is proportional to its abundance. Detection of the living rare population shown in the present results is consistent with the studies in freshwater lakes^21^, glacier-fed streams^26^, open ocean^27^, and costal ocean^28,29^. This also suggests that the metabolically active rare biosphere may be more ubiquitous than ever thought before, since it has been observed in various distinct ecosystems such as aerobic, anaerobic, freshwater, marine, surface, and subsurface environments in our present study.

In general, both deterministic niche specialization processes and stochastic neutral processes contribute to microbial community assembly, and relative contributions of these two processes to community assembly vary by the communities^30–32^. At subcommunity levels, a structure of abundant subcommunity would be more correlated to environmental factors (i.e. deterministic process) because the present environmental conditions are favorable for the growth of dominant species^33^, whereas rare subcommunity is rather considered to be randomly or stochastically shaped (i.e. neutral process)^1^. This notion provokes an assumption that community structure of rare populations would be less determined by environmental factors and consequently less correlated to the variation of environmental conditions than that of abundant populations.

Contrary to this assumption, we observed that the rare populations of groundwater community exhibited a clear sample clustering pattern and a correlation with geochemistry similar to those observed in the entire community based on community structures (Fig. 5). Only a few studies have reported a non-random community assembly of the rare biosphere and pointed out an involvement of deterministic factors therein^34–36^, but the relevant determinants were rarely identified^16^. Our results indicate that geochemical variables could influence the deterministic assembly processes of the rare biosphere. Since there was no clear distance-decay relationship in the community structure for either the entire community or the abundant and rare subcommunities (Supplementary Fig. S8), the observed differentiation of community structure did not result from simple neutral processes such as stochastic dispersal^37^, and rather implicates that geochemical factors are likely contributing more to the community assembly. Given that an aquifer system is, in general, an environment that does not fluctuate much^17^, a large part of rare populations are likely permanent members of the community, not drifters who temporally exist there, implicating that their community structure has deterministically shaped over long time scales under stable environmental conditions.

In our results, geochemical variables which highly contributed to community differentiation were sulfate, methane, NVOC, ammonium, iron, and manganese (Fig. 5CD), and the community differentiation was explained by correlating the geochemistry and the inferred function of species characteristic to each community group. Key features in the distribution pattern of microbial clades were shared by the entire community and the rare subcommunity, such as the distribution trend of *Proteobacteria* and the dominance of sulfate reducing bacteria (SRB) in Group III among the clades contributing to community differentiation, indicating that similar abiotic factors partly govern the assembly processes of the entire community and the rare subcommunity^35,38^.

Sulfate concentrations separated Group III from Groups I and II (Fig. 5CD). It has been suggested, based on bedrock geology and δ^34^S data, that the source of high sulfate concentrations in the northeastern area where Group III samples were obtained is pyrite oxidation and dissolution of sulfate minerals existing in pyritic coals and shales of the bedrock^15,16^. This high concentration of sulfate very likely attributed to a large population size of SRB belonging to *Nitrospira*, *Clostridia*, and *Deltaproteobacteria* in Group III (Supplementary Fig. S3), which were detected in both the DNA- and the RNA-based community analyses. Likewise, SIMPER analysis of the rare subcommunity detected a large proportion of SRB belonging to *Deltaproteobacteria* (*Desulfovibrionales* and *Syntrophobacterales*; Supplementary Fig. S7) in Group III as high-contributing taxa. These results showed that sulfate is a key determinant for community differentiation in the Mahomet Aquifer in accordance with previous studies^19,39^.

Conversely, negligible sulfate concentrations and high methane concentrations were observed in Group I (Supplementary Table S2). The western region where Group I samples were obtained has primarily shales with coal seams bedrock and harbors a large amount of glacial till including organic-rich paleosols and peat deposits, resulting in negligible sulfate and a substantial amount of organic carbon^18,19^. Since methanogens often compete for electron donors with SRB, negligible sulfate concentrations likely limited the dominance of SRB and enabled methanogens to proliferate, as is often observed in various anoxic environments^40^. Indeed, methanogens comprised from 71 to 89% of the total archaeal population of each Group I sample (Fig. 3B) and contribute to the emission of large amounts of methane^19,41^. Though there is no recent study investigating methane emission rates from the Mahomet Aquifer, methane production from glacial-gas wells drilled in the northeastern part of Illinois including the Mahomet Aquifer area is about 1.5 m^3^/min on average and can reach about 100 m^3^/min^42,43^. Methane analyzed in our study is of biological origin, as indicated by stable isotope signatures (Supplementary Table S2; δ^13^C values between −90‰ to −60‰ and δD values between −240‰ to −160‰), in agreement with what Hackley *et al*.^19^ reported for the Mahomet Aquifer. Most parts of methanogenic clades detected in this study belong to hydrogenotrophic methanogenic groups (e.g. *Methanobacteriales*, *Methanomicrobiales*), and acetoclastic methanogenic groups (e.g. *Methanosarcinaceae*, *Methanosaetaceae*) were less detected, indicating that the hydrogenotrophic methanogens mainly contribute to methane emission (Fig. 3B and Supplementary Fig. S3). Although methanogens were clearly found to be characteristic taxa in Group I in SIMPER analysis for the entire community, it was not the case for the rare subcommunity (Supplementary Fig. S7), perhaps because methane production might be achieved by the limited number of abundant or intermediately-abundant (0.1 to 1% relative abundance) species so that methanogens were not detected in SIMPER analysis for the rare subcommunity. A high methane concentration likely leads to a proliferation of methanotrophic clades in groups such as *Crenothricaceae* and *Methylocystaceae* (Supplementary Fig. S3), indicating that community assembly process is governed not only by geochemical variables having a geological origin but also by factors resulted from microbial activities.

In addition to methane, NVOC and ammonium explained separation of Group I from Groups II and III (Fig. 5CD); a relatively large amount of organic carbon and nitrogen could enhance the activity of heterotrophic bacterial populations. However, NVOC and ammonium can be utilized by very broad range of heterotrophic microorganisms, so that it is very hard to identify any specific clades which are primarily affected. Nevertheless, the slight increase of total cell number in Group I samples might indicate the effect of high NVOC and ammonium concentrations on total biomass production (Supplementary Fig. S9).

Iron and manganese were shown to contribute to the separation of Group I from Groups II and III and Group II from Groups I and III, respectively (Fig. 5CD). Since iron- and manganese-utilizing microorganisms are widely distributed in the natural environments and known to play key roles in natural microbial communities^44–46^, these metals can be key variables to determine the community structure in the aquifer ecosystem. Although some clades of Fe- and/or Mn-utilizing microorganisms were detected in relatively Fe and/or Mn rich sites in our analysis (e.g. *Gallionellaceae*, *Geobacteraceae*) (Supplementary Fig. S3), it is difficult to identify other specific clades involving iron and/or manganese metabolisms using the present datasets, since iron- and/or manganese-utilizing bacteria are broadly distributed within diverse phylogenetic clades^44–46^. More detailed analyses focusing on dissimilatory iron and manganese metabolisms (e.g. targeted isolation, diversity analysis based on manganese oxidizing/reducing genes) are needed for deeper understandings.

At present, it is hard to speculate on the relationships between the geochemical variables and the representative microbial clades in detail due to the dominance of clades which consist of functionally diverse members and the limited information about the physiology of diverse functionally unknown clades. It is obvious especially in the rare subcommunity. Functional information of uncharacterized species, which can be obtained by isolation and/or metagenomic approaches, will deepen our understanding for overall relationships between the microbial diversity and the geochemical profiles.

We revealed the structure of the living microbial community including the rare biosphere in the Mahomet Aquifer system at a high resolution by using massive sequencing techniques. Deep sequencing analyses uncovered the community structure, and combining these with geochemical analyses revealed the correlation between community structure and groundwater geochemistry, implying the importance of deterministic processes for community assembly of the rare biosphere. The presence of living rare biosphere having a wide variety of metabolisms and niches indicates a potential of uncontaminated subsurface groundwater ecosystems to cope with environmental deterioration, as observed in many cases of contaminated aqueous environments^47,48^. Our results are a rare example of not only deeply clarifying the microbial community structure in an aquifer system but also describing properties of the living rare biosphere and correlating abiotic parameters in terrestrial subsurface environments. Expanding our knowledge about the structure and function of the rare biosphere leads to a deeper understanding of microbial community and functional dynamics in diverse natural environments.

## Methods

### Sampling site and sample collection

The Mahomet Aquifer is a glacial aquifer composed of sands and gravels derived from glacial outwash by Pleistocene glaciations in the Mahomet bedrock valley in east-central Illinois. Aquifer sediments are interbedded with confining layers of glacial till which consist of silt, clay, paleosols, and peat deposits^18,19^. Groundwater samples were collected during October to November of 2011 from 13 sampling points in the Mahomet Aquifer, including both monitoring wells maintained by the Illinois State Geological Survey (ISGS) and municipal water wells (Fig. 1). The bedrock strata underlying the sampling area are as follows^18,19^; the bedrock in the northeastern region (Onarga Valley) consists of carbonates, shales, sandstones, and coals, that in the central region mainly consists of carbonates, and that in the western region mainly consists of shales and coals. Standard procedures were used to collect water samples^19^. Briefly, the groundwater was pumped out and kept running for at least 40–60 min while physicochemical parameters were monitored (water temperature, pH, dissolved oxygen [DO], specific conductance [SpC], and oxidation-reduction potential [ORP]). Once these parameters stabilized, water samples for geochemical analysis were passed through a 0.45 µm filter capsule and collected in Nalgene or glass bottles, acidified in the field if needed, and stored at 4 °C until analysis. Microbial cell samples were harvested on-site by filtering approximately 60–80 L of groundwater with 0.22 μm pore size mixed cellulose esters membranes (90 mm; MF-Millipore™ Membrane Filters, Merck, Darmstadt, Germany). Filtered membranes with cells were immediately transferred to 50 mL conical polypropylene tube in dry ice, and then transferred to the laboratory and stored at −80 °C until use. Basic information for each well and groundwater chemistry are shown in Supplementary Table S1.

### Analyses of geochemical and isotopic profile and total cell numbers

Cations, anions, non-volatile organic carbon (NVOC), methane (CH_4_), and the stable isotopic signatures of CH_4_ (δD and δ^13^C) and dissolved inorganic carbon (DIC) (δ^13^C) were measured at the Illinois State Water Survey (ISWS) and ISGS laboratories (Champaign, IL, USA) using standard methods (details are shown in Supplementary Materials) described by Hackley *et al*.^19^. Total cell numbers were measured by a direct count method. Briefly, cells trapped on 0.22 μm pore size Isopore^TM^ membrane (Merck, Darmstadt, Germany) were stained with 1 μg/mL 4′,6-diamidino-2-phenylindole (DAPI) and counted under Axio observer epifluorescent microscope (Carl Zeiss, Oberkochen, Germany). Cell counts were performed with more than three replicates for each sample.

### DNA/RNA extraction

Total DNA and RNA were extracted from filtered groundwater samples using methods described by Schmidt *et al*.^49^ with modifications. Briefly, the filter was cut with a sterile razor, and a part of the cut filter was then transferred into Lysing Matrix E (MP Biomedicals, Santa Ana, CA, USA). After adding DNA extraction buffer (0.1 M Tris-HCl, 0.1 M ethylenediaminetetraacetic acid, 0.75 M sucrose), cells in the filtered sample were physically disrupted by bead beating and then enzymatically and chemically lysed by lysozyme (1 mg/mL), achromopeptidase (0.01 mg/mL), proteinase K (0.1 mg/mL), and sodium dodecyl sulfate (1% [w/v]). The nucleic acid fraction was extracted by cetyl trimethyl ammonium bromide (1% [w/v]) and chloroform-isoamyl alcohol (24:1). Extracted nucleic acids were precipitated with isopropanol and washed with ethanol, and then fractionated into DNA and RNA by ALLPrep DNA/RNA mini kit (Qiagen, Hilden, Germany), according to manufacturer’s instructions. DNA and RNA samples were treated with RNase and DNase, respectively, to remove contaminants. Removal of DNA contamination from the RNA samples was confirmed by PCR amplification. DNA and RNA concentrations were spectrometorically measured using a Nanodrop 2000c (Thermo Scientific, Wilmington, DE, USA).

### Construction of 16S amplicon library

A DNA-based 16S amplicon library was constructed by PCR amplification of target regions (V4) of 16S rRNA genes with specific primers, which is a commonly used hypervariable region in environmental microbial community analyses because it can detect a wide range of bacterial and archaeal taxonomic clades^50–55^. Primers used for amplification, multiplexing, and sequencing were based on 515 F and U806R, according to the original protocol in Earth Microbiome Project (http://press.igsb.anl.gov/earthmicrobiome/protocols-and-standards/16s/) and previously described methods^56^. PCR was performed using AmpliTaq Gold LD (Applied Biosystems, Foster City, CA, USA) according to manufacturer’s instructions with the following program: initial denaturation at 95 °C for 2 min, followed by 30 cycles of 95 °C for 30 s, 50 °C for 30 s and 72 °C for 2 min with a final extension at 72 °C for 5 min. Template DNA mass was 1 or 0.2 ng per tube. Each sample was amplified in pentaplicate to avoid molecular sampling error and pooled into one after the reaction.

Two-step RT-PCR was performed for construction of a RNA-based 16S amplicon library. cDNA samples were generated by RT-PCR using ReverTra Ace alpha (TOYOBO, Osaka, Japan) according to manufacturer’s instruction with the reverse primer U806R (5′-GGACTACHVGGGTWTCTAAT-3′). Specific PCR targeting 16S rRNA genes with non-RT control samples indicated no genomic DNA contamination in the cDNA samples. PCR amplification from the cDNA samples was performed as described in DNA-based library preparation with a change in the cycle number at amplification step to 20 cycles.

The obtained PCR products were purified by Agencourt AMPure XP kit (Beckman Coulter, Brea, CA, USA), and then fluorometrically quantified by Qubit and Quant-iT High-Sensitivity DNA Assay Kit (Invitrogen, Carlsbad, CA, USA). Quality control was performed with Bioanalyzer (Agilent, Santa Clara, CA, USA) using DNA1000 kit (Agilent) to check the purity of the amplicon, resulting in detection of a single peak of target products in all samples. Twenty-six samples (one half DNA-based amplicons and other half RNA-based amplicons) were pooled at even concentrations to obtain the amplicon library. Parallel massive sequencing was performed by Illumina MiSeq sequencer (Illumina, San Diego, CA, USA) with MiSeq Reagent Kit v2 (Illumina) as described previously^57^.

### Sequence data analysis

After checking read quality, each 150 bp pair-end read data was merged by PANDAseq algorithm to generate average 251 bp merged read^58^. Phylogenetic analysis of the obtained paired-end read was performed by QIIME ver. 1.5.0^59^. All reads were assembled into OTUs at 97% sequence similarity. Taxonomic assignment was conducted by using BLAST with the Greengenes database ver. 13_5^60^.

### Statistical analyses

Resemblance analysis of geochemical profile (cluster analysis) and other analyses related to microbial community composition and similarity (Bray-Curtis similarity-based multi-dimensional scaling [MDS] plot, analysis of similarity [ANOSIM], and similarity percentages [SIMPER]) were all performed by using Primer ver. 6.1.13 (Primer-E Ltd., Plymouth, UK). Cluster analysis of geochemical profiles was applied to a matrix of pairwise comparison based on Euclidian distance after data normalization and calculated by a complete linkage method.

Species abundance data used for community composition and similarity analyses were first rarified to a read number 24,741, which is 75% of the minimum obtained read number among sequenced samples (36,322), by QIIME. Abundance data for subcommunities were produced by picking up and combining all OTUs having the read number >1.0% and <0.1% of a rarified total read number of each sample for abundant and rare species, respectively^33,35^.

ANOSIM is a permutation-based statistical analysis using the unweighted UniFrac distance matrix or the Bray-Curtis similarity-based community resemblance matrix and was used to test a null hypothesis that there are no differences among the groups. The analysis produces a test statistic R ranging from -1 to 1 with a significance level (P), and a near-zero R value implies no differences between samples.

SIMPER test was applied to community composition data to identify clades contributing the differences among the groups. The average Bray-Curtis dissimilarity (AvDiss) between all pairs of samples and the contribution (Contrib%) of each clade to total dissimilarity between the groups were calculated. Higher values of the AvDiss and Contrib% indicate a higher contribution of the clade to the group discrimination.

Beta-diversity analysis was performed by QIIME based on the unweighted UniFrac distance matrix. Jackknife resampling at the depth of 24,741 was performed for generating the UniFrac-based principal coordinate analysis (PCoA) plot.

Redundancy analysis (RDA) and canonical correlation analysis (CCA) were applied for visualizing a correlation between community composition and geochemical parameters and performed using CANOCO for Windows ver. 4.5^61^ with the geochemical data and the rarified species abundance data mentioned above.

## Supplementary information

Supplementary Materials

## Data Availability

Sequence data have been submitted to the DDBJ/EMBL/GenBank databases under accession number DRA006033.
