# Supplementary material for: Diversity and geochemical community assembly processes of the living rare biosphere in a sand-and-gravel aquifer ecosystem in the Midwestern United States
Source: Sci Rep. 2019 Sep 17;9:13484. doi: 10.1038/s41598-019-49996-z (PMC6748922; doi:10.1038/s41598-019-49996-z)
Supplement: Supplementary file 1 — Supplementary Materials [file 41598_2019_49996_MOESM1_ESM.pdf]

Diversity and geochemical community assembly processes of the living rare biosphere in a sand-and-gravel aquifer ecosystem in the Midwestern United States

Kyosuke Yamamoto, Keith C. Hackley, Walton R. Kelly, Samuel V. Panno, Yuji Sekiguchi, Robert A. Sanford, Wen-Tso Liu, Yoichi Kamagata, and Hideyuki Tamaki

Table S1. Basic information of sampling well and groundwater sample and read number obtained by 16S amplicon sequencing.

Sample #	lat	lon	Depth (m)	Temp. (°C)	pH	SpC (μS cm^{-1})	ORP (mv)	DO (mg l^{-1})	Total cell count ± SD (cell ml^{-1})	Obtained read number DNA	RNA
1	40.21223671	-88.98082158	111.3	13.6	7.02	933	58	0.10	1.53 ± 0.27 10^6	74 221	51 623
2	40.07773963	-88.82327729	76.2	13.9	6.97	850	63	0.10	3.48 ± 0.36 10^6	88 804	61 584
3	39.94506437	-88.59601878	-	13.5	6.70	2,130	91	0.15	1.04 ± 0.15 10^6	87 103	67 614
4	39.9636176	-88.61311089	-	12.9	7.05	1323	54	0.14	1.30 ± 0.21 10^7	79 080	52 474
5	40.36227429	-88.13763022	97.5	14.0	6.95	586	89	0.57	3.41 ± 0.90 10^5	88 261	44 480
6	40.29799175	-88.25078176	93.6	13.5	7.28	637	102	0.10	1.17 ± 4.60 10^7	78 926	52 266
7	40.24653607	-88.30680191	94.0	13.9	7.24	607	95	0.10	8.58 ± 1.15 10^5	90 768	65 439
8	40.05320026	-88.55916074	88.4	13.1	6.77	940	106	0.10	4.66 ± 0.67 10^5	77 759	50 166
9	40.10650066	-88.5119468	61.0	12.6	6.90	608	90	0.10	9.33 ± 2.48 10^5	133 990	54 465
10	40.67904711	-88.04988485	68.6	13.0	6.85	1,312	86	0.13	6.05 ± 0.54 10^5	109 389	48 092
11	40.51604715	-87.7064742	70.7	13.9	6.95	705	227	0.22	1.50 ± 0.17 10^6	90 503	65 710
12	40.60211425	-87.88439957	55.5	13.8	7.00	735	60	0.22	1.20 ± 0.20 10^6	100 484	72 802
13	40.45954912	-87.76384023	73.2	13.2	7.06	742	19	0.49	5.69 ± 1.18 10^5	85 350	36 322

Table S2. Geochemical and isotopic profiles in groundwater samples.

Sample #	Environmental variables[a] (mg l^{-1})																			Stable isotopes (‰)		
	B	Ba	Ca	Fe	K	Mg	Mn	Na	P	Sr	Zn	As[b]	NH_3-N	F	Cl	SO_4	Br	NVOC[c]	CH_4[d]	$\delta^{13}C$ (DIC[e])	$\delta^{13}C$ (CH_4)	δD (CH_4)
1	0.56	0.56	60.6	1.75	2.58	29.9	0.02	112	0.368	0.45	<0.0097	28.93	3.04	0.80	75.1	<0.31	0.22	10.54	2.620	-6.0	-83.4	-235
2	0.46	0.27	69.2	1.93	2.50	39.0	0.02	73	0.422	0.56	<0.0097	10.63	2.89	0.59	34.3	<0.31	0.08	9.01	2.000	-5.2	-80.2	-223
3	0.25	0.40	100.4	3.51	1.71	39.9	0.07	286	0.303	0.56	0.031	4.35	3.25	0.49	475.4	<0.31	0.81	8.28	4.170	-2.9	-82.1	-232
4	0.54	0.30	55.5	2.08	1.95	25.2	0.03	202	0.352	0.63	<0.0097	18.49	2.10	0.75	244.2	<0.31	0.53	9.67	3.750	-7.6	-85.5	-230
5	0.27	0.05	70.9	1.14	1.69	27.6	0.03	23	0.207	0.42	<0.0097	<0.95	0.76	0.26	1.5	<0.31	<0.08	2.84	<0.01	-10.8	ND[f]	ND
6	0.23	0.05	56.4	0.46	1.79	24.4	0.13	70	0.107	0.34	<0.0097	26.09	0.59	0.36	1.2	13.6	<0.08	2.57	0.002	-8.6	-67.4	ND
7	0.22	0.09	78.4	0.76	1.91	31.2	0.07	20	0.211	0.45	<0.0097	3.55	1.23	0.31	2.5	8.3	<0.08	2.64	0.038	-9.4	-71	-192
8	0.08	0.16	127.3	2.06	1.31	43.0	0.13	15	0.081	0.23	0.010	6.25	0.45	0.20	46.6	78.9	<0.08	2.02	<0.01	-11.6	ND	ND
9	0.32	0.16	68.9	1.57	1.86	28.6	0.02	26	0.091	0.38	0.014	3.32	0.77	0.53	1.7	1.1	<0.08	2.60	<0.01	-10.5	ND	ND
10	0.92	0.02	156.8	2.19	3.09	60.5	0.04	76	<0.073	1.17	<0.0097	<0.95	1.95	0.35	9.8	471.1	0.12	3.06	<0.01	-15.4	ND	ND
11	0.80	<0.00085	0.1	<0.024	0.21	0.0	<0.0015	190	0.108	0.00	<0.0097	4.13	0.03	0.27	2.0	36.4	<0.08	2.22	<0.01	-13.5	ND	ND
12	0.31	0.10	71.5	1.05	1.67	31.3	0.04	24	0.612	0.31	<0.0097	0.97	1.31	0.22	2.3	13.7	<0.08	1.31	<0.01	-12.3	ND	ND
13	0.53	0.12	67.5	1.43	2.53	29.9	0.04	31	0.350	0.97	<0.0097	7.26	0.87	0.34	1.5	20.5	<0.08	1.76	<0.01	-11.6	ND	ND

a, Following variables were tested but under detection limits in all 13 groundwater samples: Al, Be, Cd, Co, Cr, Cu, Li, Mo, Ni, Pb, Sb, Se, Sn, Ti, Tl, V, and NO_3-N; b, µg l^{-1}; c, Non-volatile organic carbon; d, mmol l^{-1}; e, Dissolved inorganic carbon; f, Not determined

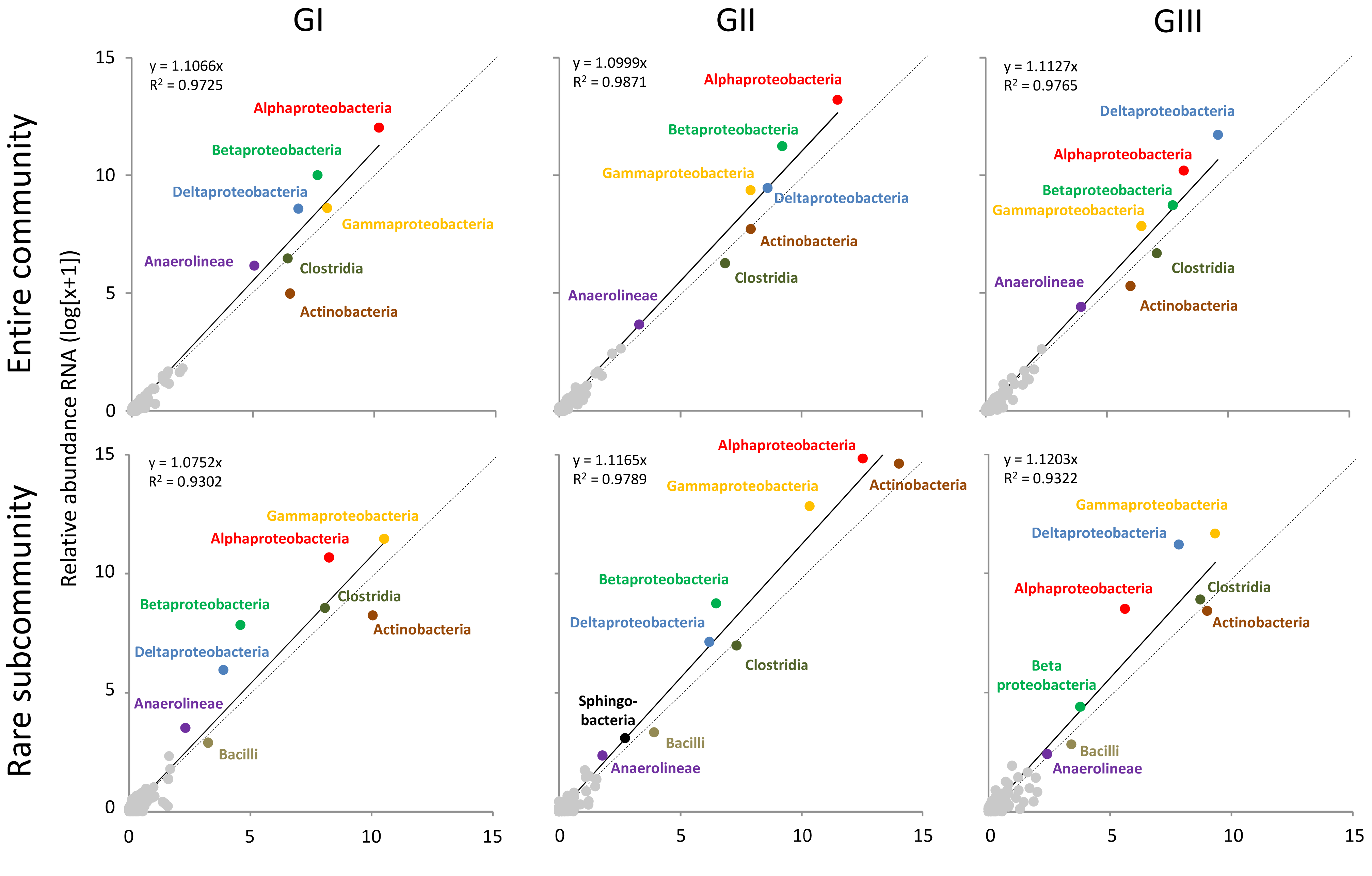

Relative abundance DNA (log[x+1])

Figure S1. Correlation of relative abundance based on the RNA- and the DNA-based community. Lines represent linear regressions. Doted lines represent slope = 1.

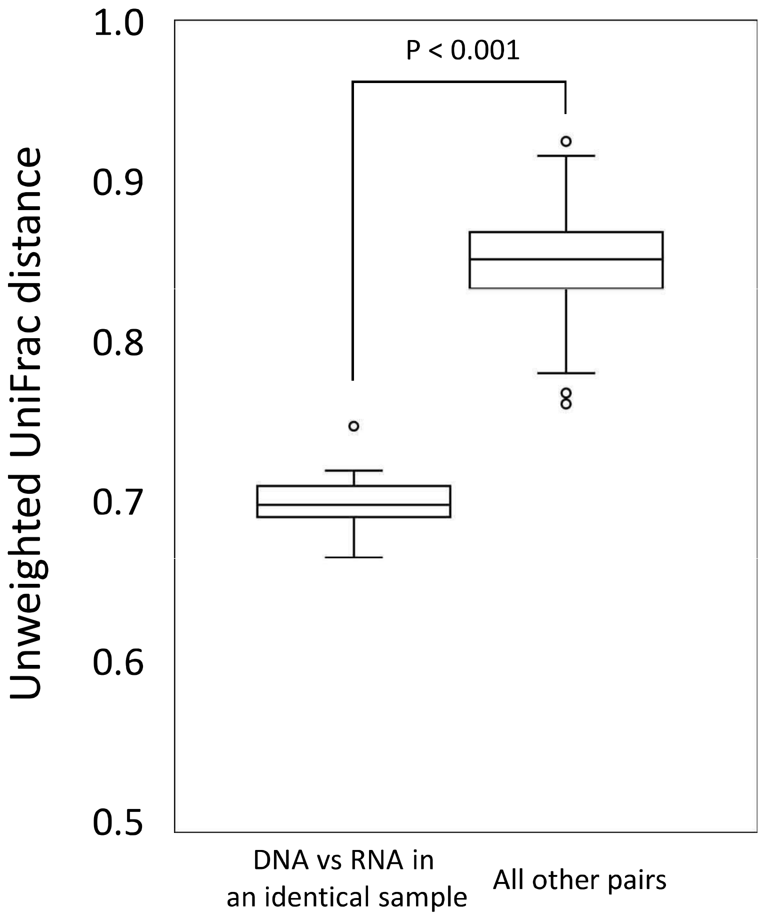

Figure S2. Dissimilarity (unweighted UniFrac distance) between subsurface groundwater communities. Distance between DNA- and RNA- based community structure in an identical sample (N=13) and distance between all other pairs (N=312) are shown.

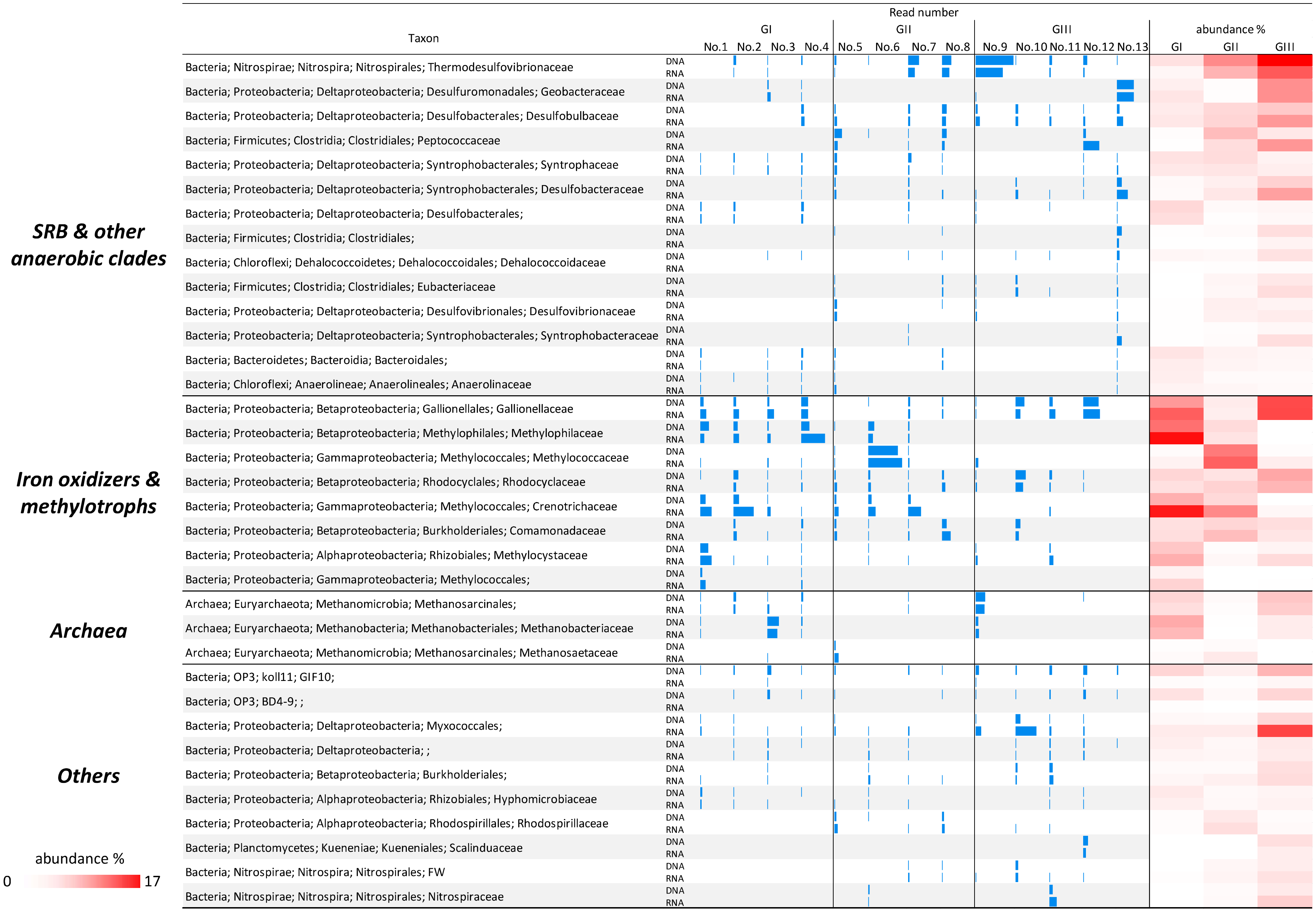

Figure S3. Distribution of dominant OTUs in the groundwater community. Blue bar indicates read number (e.g. Read number of *Thermodesulfovibrionaceae* [shown at the top row] in No. 9 was 14,513). Heat map indicates abundance percentage of each taxon in each group.

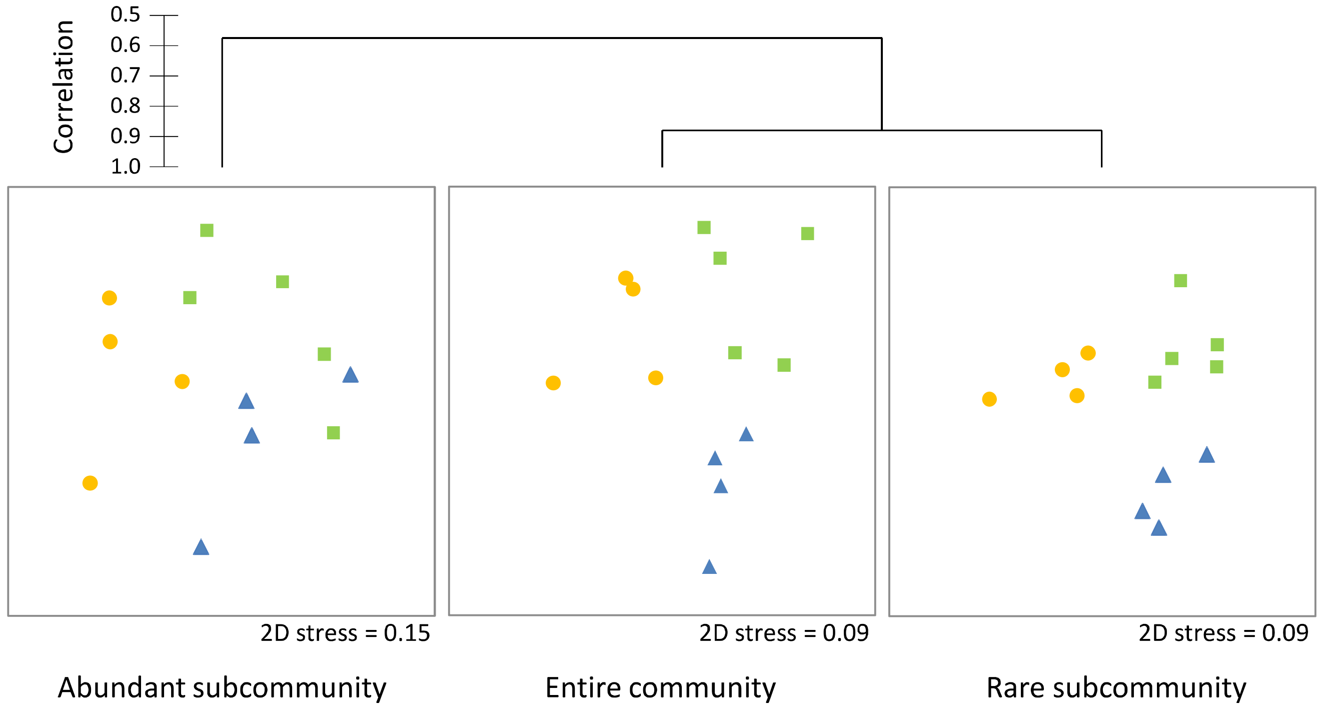

Figure S4. Comparison of community resemblance pattern between the entire community, the rare subcommunity and the abundant subcommunity. 2D MDS plot was based on Bray-Curtis similarity matrix using log-transformed abundance data (DNA-based community). Sample distribution patterns were compared using Pearson correlation analysis. Cluster analysis was performed by single linkage method. Blue triangles are Group I samples, orange circles are Group II samples, and green squares are Group III samples.

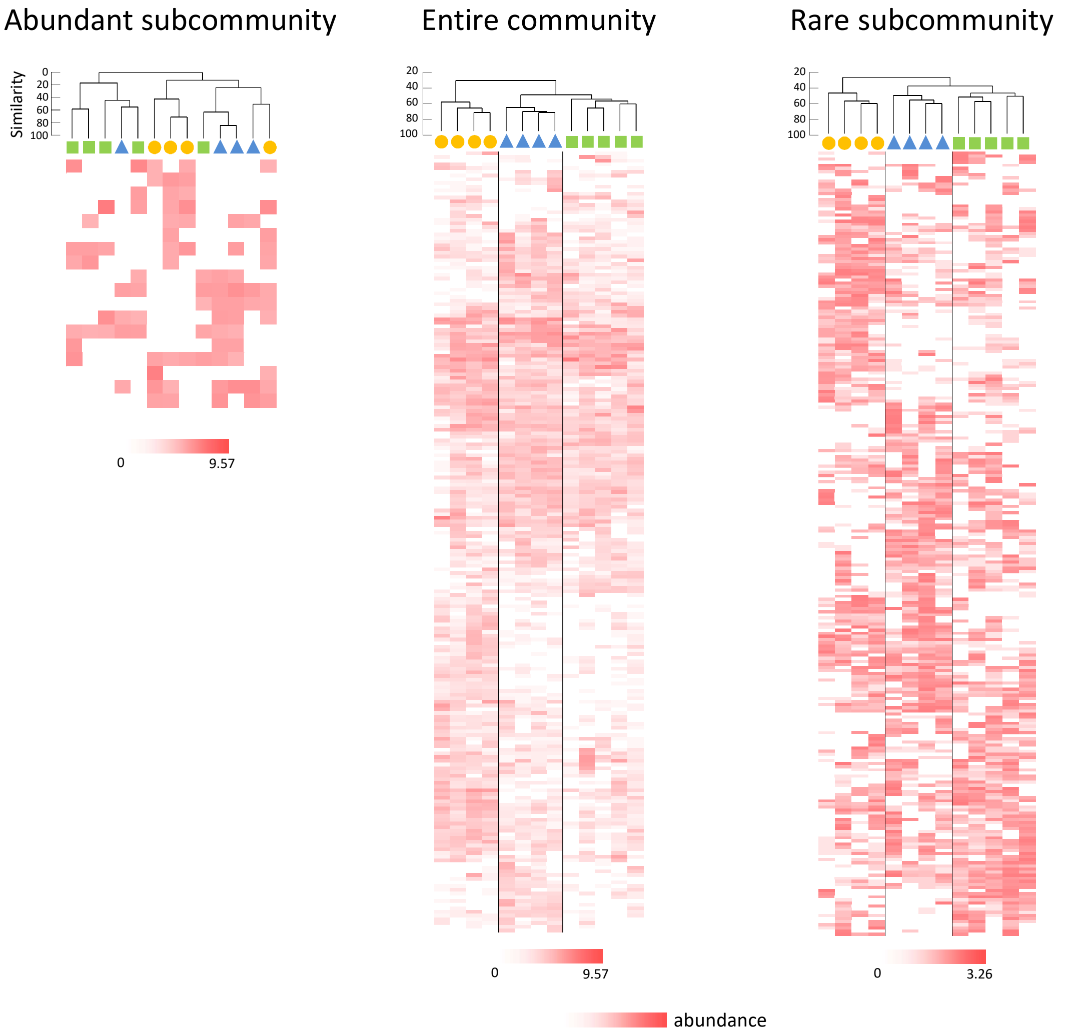

Figure S5. Abundance of OTUs highly contributing to community differentiation. High-contributing OTUs were defined as taxa ranked in the top 30% of cumulated contribution to the similarity among the groups based on similarity percentages (SIMPER) analysis. Cluster analysis was performed to group. Species abundance was expressed as a log-transformed read number. Blue triangles are Group I samples, orange circles are Group II samples, and green squares are Group III samples.

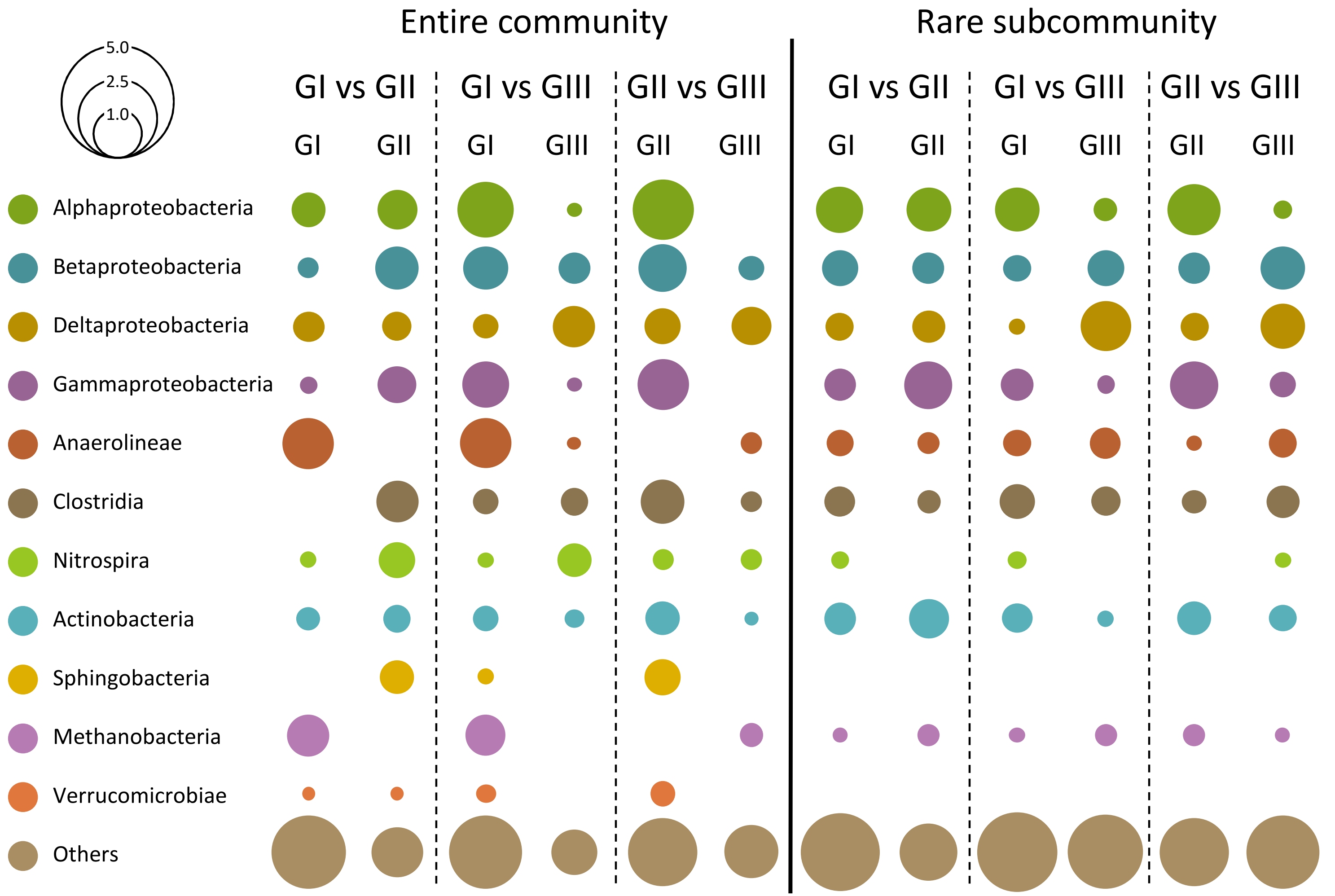

Figure S6. Composition of taxa contributing to group differentiation. Each circle represents a high-contributing taxon in the designated group, which is detected by SIMPER analysis based on DNA-based community profiles. Circle size represents proportion of contribution % of each taxon in top 30% of cumulative contribution. Each group has two circle clusters due to pairwise comparison between groups in SIMPER analysis.

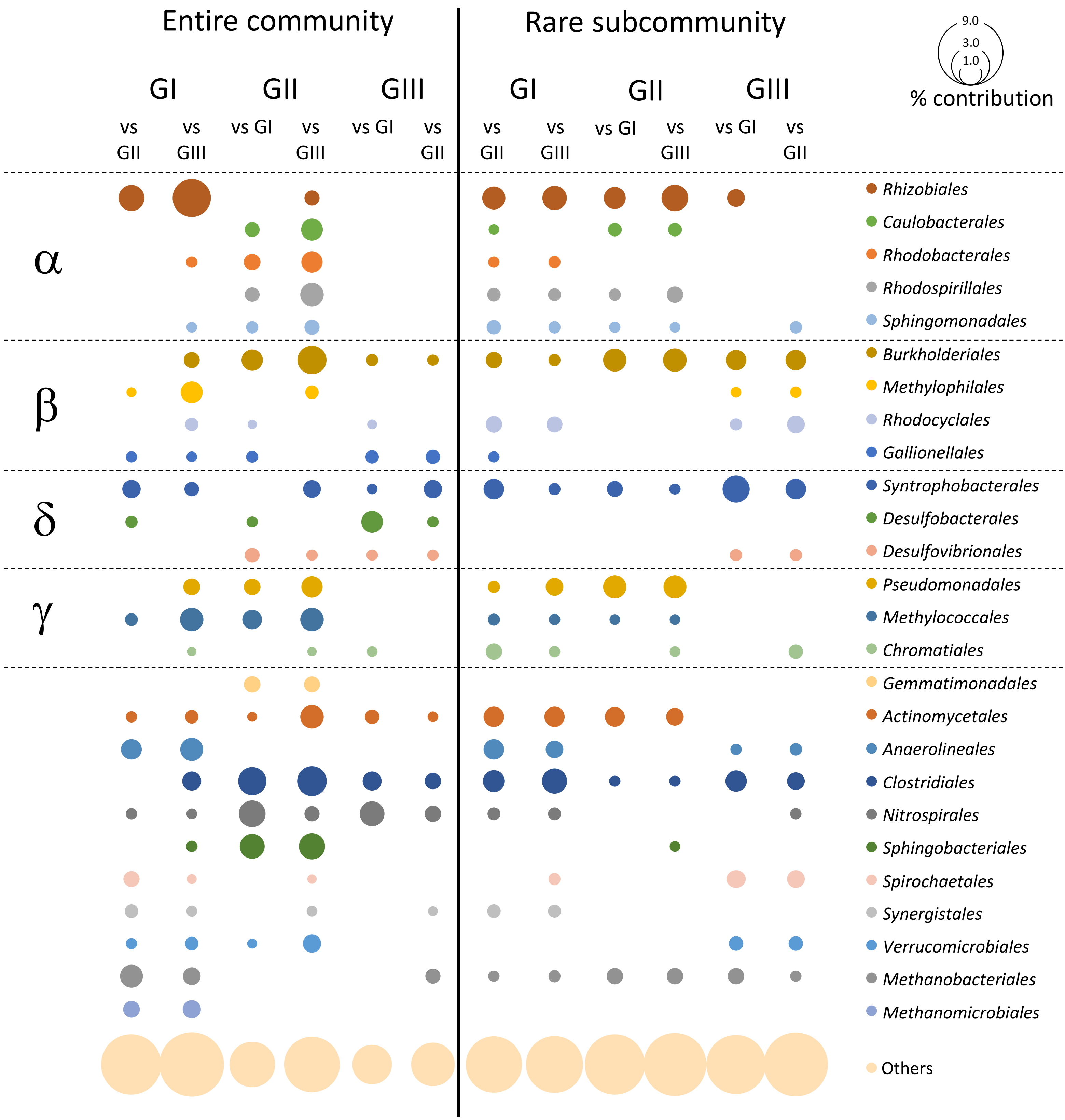

Figure S7. Composition of taxa contributing to group differentiation. Each circle represents a high-contributing taxon (Order level) in the designated group, which is detected by SIMPER analysis based on DNA-based community profiles. Circle size represents proportion of contribution % of each taxon in top 30% of cumulative contribution. Each group has two columns of circle clusters due to pairwise comparison between groups in SIMPER analysis.

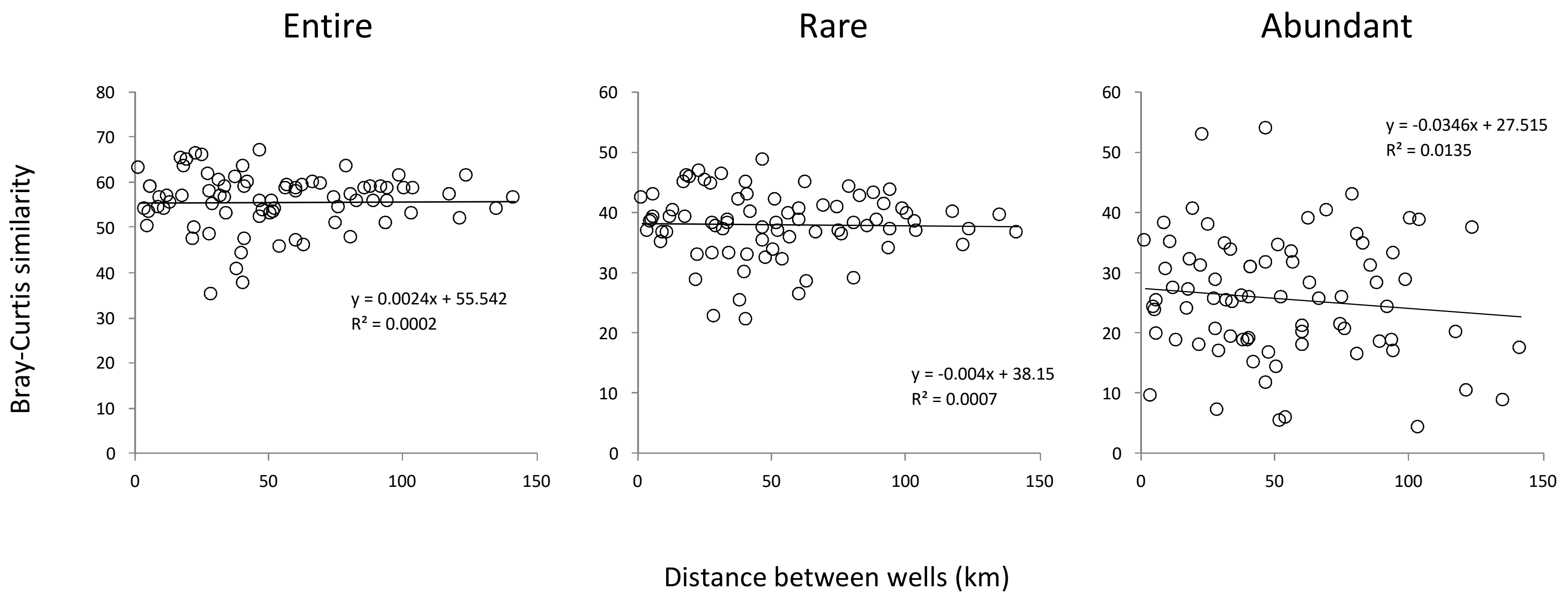

Figure S8. Distance-decay relationships. Bray-Curtis similarity and distance between sample pairs of all combination of 13 wells (n=78). Similarity was calculated using log-transformed data (log[x+1]).

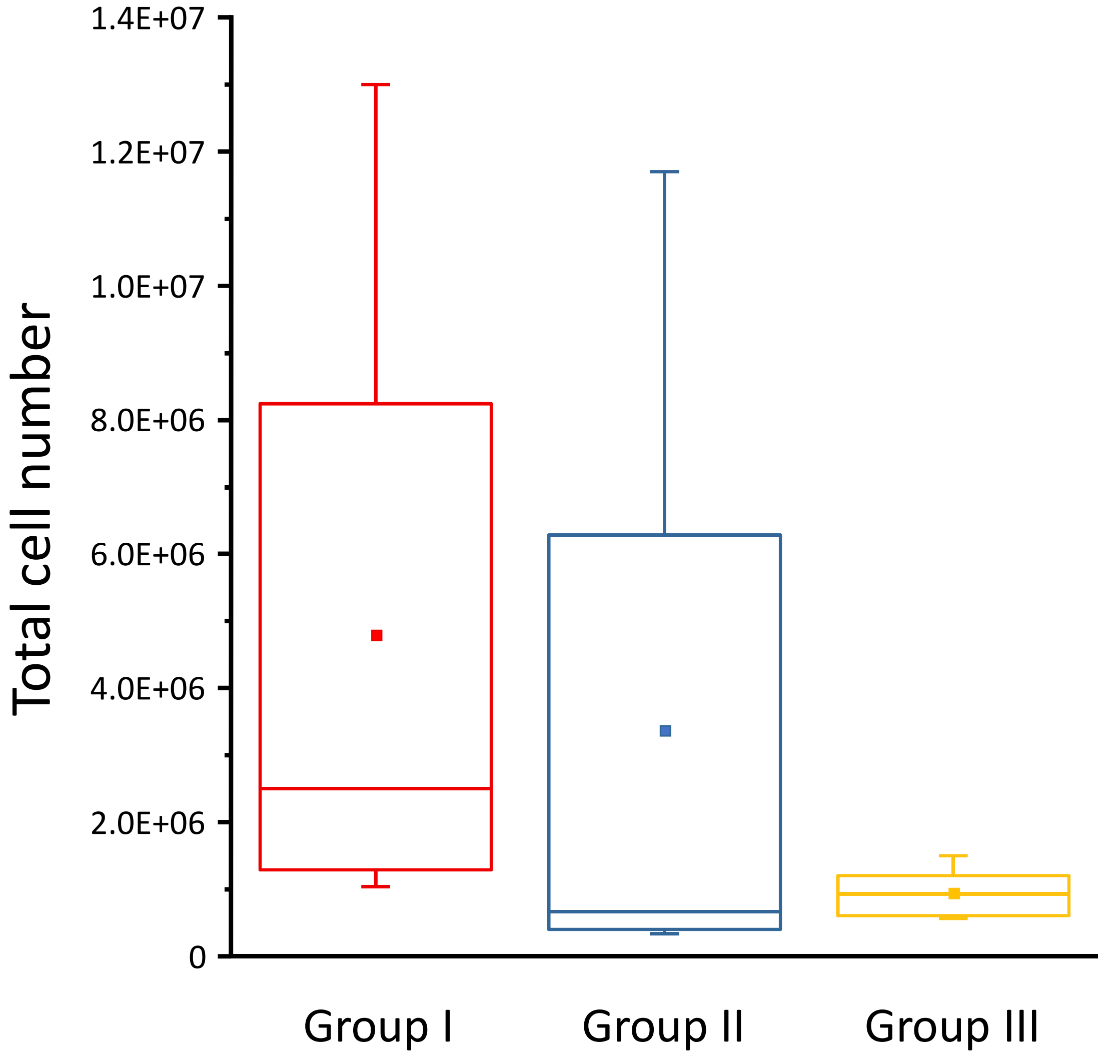

Figure S9. Total cell number of samples in each group. Boxes represent the interquartile intervals from the 25th to the 75th percentiles. Filled boxes and solid horizontal lines through the boxes represent the means and the medians, respectively. The minimum and maximum values are represented by the capped vertical bars.

Analytical methods for geochemical variables

Complete inorganic chemistry and NVOC analyses were conducted at the Illinois State Water Survey (ISWS) Public Service Laboratory (Champaign, IL) using standard analytical procedures. Anions were analyzed following U.S. EPA Method 300, using a Dionex ICS-5000 ion chromatograph (25-microliter injection loop) with a conductivity detector, an AS-DV automated sampler, and an AERS 500 eluant suppressor. Separations were carried out isocratically on an IonPac AS14 analytical column, with an AG14 guard column, using a mixture of 1.0 mM sodium bicarbonate and 3.5 mM sodium carbonate as an eluant.

Cations/metals were analyzed following U.S. EPA Method 200.7. Analyses were performed using a Varian Vista Pro CCD simultaneous inductively coupled plasma optical emission spectrometer (radial torch configuration) with an SPS 3 autosampler. Field acidified samples were digested in 2% nitric acid and 5% hydrochloric acid prior to analysis. The digested samples were nebulized for transport into the radio frequency ICP, where each of the elements emits a specific spectrum. Wavelength intensities were measured by the photosensitive CCD micro-chip.

Alkalinity was determined following Standard Method 2320B, determined by titrating 40 mL of a water sample with a standard solution of 0.02 N sulfuric acid to an endpoint of pH 4.50. The procedure was automated, using a Mettler Toledo T70 titrator, a pH combination electrode, and a Rondo autosampler.

NVOC was determined following Standard Method 5310B, using a Shimadzu TOC-L total organic carbon analyzer, an ASI-L autosampler, and TOC-Control L software. Organic carbon in a sample was converted to carbon dioxide (CO_2) by catalytic combustion, and the CO_2 formed was measured directly by a nondispersive infrared detector.

Ammonia-nitrogen was determined following U.S. EPA Method 350.1, using automated colorimetry. Reagents and samples were mixed and analyzed using a Lachat QuikChem 8500 Series 2 Flow-Injection Analyzer with Omnion software. Ammonia reacts with alkaline phenol and hypochlorite to form indophenol blue. The blue color was intensified with sodium nitroferricyanide, and absorbance was measured at 630 nm.

Arsenic was determined following U.S. EPA Method 200.9, using an Agilent Technologies 240Z Graphite Furnace Atomic Absorption Spectrometer, with Zeeman background correction, a PSD 120 Programmable Sample Dispenser, and SpectAA software. Samples acidified in the field were digested in 1% nitric acid prior to analysis.

Groundwater samples taken for methane gas determination were collected in 1 gallon (4 L) collapsible containers having caps that were fitted with plastic spigots. The containers were evacuated in the field using a portable directdrive pump. Thirty mL of 0.13% Zephrin chloride solution, a preservative, was added to the containers prior to evacuation. The collapsible containers were then immediately connected to the Viton tubing via the spigot in the cap. Unfiltered water was allowed to flush the connecting tubes and spigot for several seconds to rid the system of air bubbles, and then the valve was opened to collect the water sample. The sample container was filled with slightly less than one gallon of water and brought back to the laboratory for processing that same afternoon. These large samples were not kept chilled.

The concentration of dissolved CH_4 was determined by analyzing the composition of the gas bubble from the 4 L collapsible container and using a best-fit polynomial for CH_4 solubility data between 0 and 30 °C (Dean, 1992) to calculate the concentration of CH_4. The sample containers were brought back to the laboratory and weighed immediately. The quantity of water was determined from the difference between the full and empty weights of the collapsible sample containers. By the time the sample was returned to the laboratory, the dissolved gases had equilibrated to atmospheric pressure and come out of solution, making a bubble inside the container. The gas was extracted from the containers that same day using an appropriate-size graduated syringe and needle. Prior to extracting the gas, saturated sodium sulfate solution was used to fill the needle and dead space at the end of the syringe in order to minimize air contamination of the samples and prevent dissolution of the gas sample into the solution while in the syringe. The gas was extracted by pushing the needle directly through the plastic collapsible container and drawing the gas bubble into the syringe. The quantity of gas extracted was measured using the graduated marks on the syringe and was injected into a previously evacuated glass vial (Vacutainer®) fitted with a septum. The gas samples were then analyzed on a gas chromatograph (GC).

Stable isotopic analyses included $\delta^{13}C$ of dissolved inorganic carbon (DIC), and $\delta^{13}C$ and δD of the CH_4. The $\delta^{13}C$ and δD values of the CH_4 samples were determined by combusting the CH_4 and collecting the products CO_2 and H_2O as described by Hackley et al. (1999).

The δD and $\delta^{13}C$ values were determined on a dual inlet ratio–mass spectrometer. Each sample was directly compared to an internal standard calibrated versus an international reference standard. The final results are reported versus the international reference standards. The δD results are reported versus the international Vienna Standard Mean Ocean Water (V-SMOW) standard. The $\delta^{13}C$ results are reported versus the Peedee belemnite (PDB) reference standard. Analytical reproducibility for δD and δ13C were equal to or less than ±1.0‰ and ±0.15‰, respectively.

Table 1. Instrument Detection Limits (mg/L).

Parameter	Detection Limit
As	0.00079
B	0.023
Ba	0.00085
Ca	0.029
Fe	0.024
K	0.016
Mg	0.027
Mn	0.0015
Na	0.026
P	0.073
Sr	0.00037
Zn	0.0097
alkalinity	4
F^-	0.07
Cl^-	0.16

Br^-	0.08
NO_3 -N	0.04
SO_4^{2-}	0.21
NVOC	0.31
NH_3-N	0.03

Dean, J.A., 1992, Lange's Handbook of Chemistry (14th ed.): New York, McGraw-Hill.

Hackley, K.C., Liu, C.L., and Trainor, D., 1999, Isotopic identification of the source of methane in subsurface sediments of an area surrounded by waste disposal facilities: Applied Geochemistry, v. 14, p. 119–131, doi: 10.1016/S0883-2927(98)00036-5.
